# Imaging genomics of cancer: a bibliometric analysis and review

**DOI:** 10.1186/s40644-025-00841-9

**Published:** 2025-03-04

**Authors:** Xinyi Gou, Aobo Feng, Caizhen Feng, Jin Cheng, Nan Hong

**Affiliations:** 1 Department of Radiology, Peking University People’s Hospital, Beijing, China; 2 College of Computer and Information, Inner Mongolia Medical University, Inner Mongolia, China

**Keywords:** Imaging genomics, Cancer, Bibliometric analysis, Review

## Abstract

**Background:**

Imaging genomics is a burgeoning field that seeks to connections between medical imaging and genomic features. It has been widely applied to explore heterogeneity and predict responsiveness and disease progression in cancer. This review aims to assess current applications and advancements of imaging genomics in cancer.

**Methods:**

Literature on imaging genomics in cancer was retrieved and selected from PubMed, Web of Science, and Embase before July 2024. Detail information of articles, such as systems and imaging features, were extracted and analyzed. Citation information was extracted from Web of Science and Scopus. Additionally, a bibliometric analysis of the included studies was conducted using the Bibliometrix R package and VOSviewer.

**Results:**

A total of 370 articles were included in the study. The annual growth rate of articles on imaging genomics in cancer is 24.88%. China (133) and the USA (107) were the most productive countries. The top 2 keywords plus were “survival” and “classification”. The current research mainly focuses on the central nervous system (121) and the genitourinary system (110, including 44 breast cancer articles). Despite different systems utilizing different imaging modalities, more than half of the studies in each system employed radiomics features.

**Conclusions:**

Publication databases provide data support for imaging genomics research. The development of artificial intelligence algorithms, especially in feature extraction and model construction, has significantly advanced this field. It is conducive to enhancing the related-models’ interpretability. Nonetheless, challenges such as the sample size and the standardization of feature extraction and model construction must overcome. And the research trends revealed in this study will guide the development of imaging genomics in the future and contribute to more accurate cancer diagnosis and treatment in the clinic.

## Advances in knowledge

Imaging genomics is considered a crucial component in cancer precision medicine. A review is necessary to offer a holistic and systematic view of this field.

Focused on survival and classification, imaging genomics shows potential in cancer heterogeneity, yet faces challenges on standardization, model complexity, and ethics.

This study clarifies the objectives, current standing, and evolution of imaging genomics in cancer, offering a basic workflow and trends for cancer analysis in this domain.

## Background

Cancer remains a devastating global health challenge, exerting a significant and escalating toll on societies and healthcare systems worldwide. The Global Cancer Statistics 2022 report reveals that in 2022, nearly 20 million individuals were diagnosed with cancer, resulting in 9.7 million cancer-related deaths [1].

Diagnostic imaging techniques such as Computed Tomography (CT), Magnetic Resonance Imaging (MRI), Positron Emission Tomography (PET), and Ultrasound (US) are integral to the detection, staging, and monitoring of cancer treatment responses [2–4]. They can perform non-invasive multiple scans, providing information about the cancer and surrounding tissues, and can be compared for changes over time. With the advancement of artificial intelligence algorithms, the scope of imaging features has expanded beyond conventional imaging features, such as tumor size, shape, and margin, to encompass high-throughput, algorithm-based radiomics features [5, 6]. These mainly include handcrafted radiomic, and deep learning features, which may not be perceptible to the human eye [7].

In addition to imaging technology, advances in genomics have also provided new perspectives for cancer research, and the combination of the two has given birth to the interdisciplinary discipline of imaging genomics. Imaging genomics, also known as radiogenomics, is an emerging field that lies at the intersection of medical imaging and genomics [8, 9]. The study of genomics involves analyzing the complete genetic makeup, encompassing DNA examination, transcriptomics (which includes the investigation of mRNAs, miRNAs, and lncRNAs), and epigenomics research [10, 11]. The term radiogenomics, which first emerged in 2002, has expanded beyond its initial definition [12, 13]. Originally, it described research examining the relationship between patient genetics and variations in their sensitivity to radiation therapy. However, its content has now been expanded to include imaging genomics. The primary objective of imaging genomics is to identify and establish relationships between both image features (semantic and quantitative) with genomic information, thereby constructing association maps that can further be correlated with clinical outcomes or other relevant metrics [13, 14]. By creating a comprehensive map of associations, it can enhance the precision of prognostic and predictive models across a range of oncological conditions. This integration of imaging data with genomic insights holds the potential to improve patient stratification and treatment planning [15–17].

The underlying mechanism of imaging features can be discovered by imaging genomics, which may enhance the credibility of its clinical value and provide potential target of therapeutic intervention [18–20]. The formal classification of molecular subtypes through genomic analysis is both costly and time-consuming. In clinical practice, immunohistochemistry(IHC) methods are often employed as an alternative for molecular typing. Nevertheless, IHC specimens are typically extracted from a single localized region. Factors such as tissue preparation, tumor heterogeneity, or genetic variations affecting protein expression can result in discrepancies between IHC and genetic testing outcomes [21, 22]. Discordance rates between genetic testing and IHC classifications can indeed vary significantly, with reported rates ranging from about 40–100% across different studies [23–25]. Through imaging genomics, it becomes possible to predict molecular typing and therapeutic efficacy noninvasively.

Therefore, imaging genomics research has provided valuable insights into cancer [26]. This review synthesizes the current state of imaging genomics across various cancer types, focusing on their basic workflow and challenges. Additionally, a bibliometric analysis quantitatively assessed the trajectory and focal points of imaging genomics in cancer.

## Materials and methods

### Search strategy

Data collection was conducted utilizing the PubMed, Embase, and Web of Science databases, and was enhanced by examining the reference lists of pertinent articles included in our review up to July 2024. The search query of PubMed and Web of Science is (“imaging genomics” OR ‘radiogenomics’) AND “cancer”. For Embase, the search string is (‘imaging genomics’/exp OR ‘imaging genomics’ OR ‘radiogenomics’/exp OR ‘radiogenomics’) AND (‘cancer’/exp OR ‘cancer’). It is noteworthy that the initial objective of radiogenomics was to identify potential genomic predictors for radiation toxicity; however, this aspect was not included in the scope of the study.

### Study selection

All articles were screened back-to-back by two independent investigators (XYG and ABF) to determine whether they met our inclusion criteria. Any disputes were solved by a discussion with a third reviewer (CZF). The inclusion criteria were as followed: (1) studies focusing on cancer patients; (2) Research utilizing imaging genomics methodologies; (3) Peer-reviewed and formally published articles. And the exclusion criteria were: (1) non-human studies; (2) Articles not written in English; (3) Review.

### Data extraction

Two investigators (XYG and ABF) extracted basic study information including author, year of publication, study design, sample size, and type of cancer. Subsequently, a more detailed extraction was performed to gather insights into the imaging genomics field, such as cancer types, imaging modalities, imaging features and genetic types. Imaging features included conventional imaging features and radiomics features.

Specifically, conventional imaging features refer to characteristics that can be directly observed, measured, and simply calculated from medical imaging, such as tumor size, shape, location, and signs (for example, mismatch on T2-FLAIR), as well as values obtained from special examinations like the apparent diffusion coefficient measurements from DWI sequences and the standardized uptake value max (SUVmax) values from PET scans. Radiomics features, on the other hand, are quantitative imaging characteristics derived from computer algorithms, such as handcrafted radiomics features based on the PyRadiomics platform and features extracted using a specific deep learning algorithm [27–29].

### Bibliometric analysis

Citation data was extracted from the Web of Science and Scopus database to perform a bibliometric analysis. It involved the keywords, keywords plus, quantification of publication trends, citation patterns, and collaborative efforts within the imaging genomics of cancer research. Keywords plus is a set of terms extracted from the titles of cited documents in an article. It aims to complement the author-provided keywords, enhancing the thoroughness and precision of literature searches. Additionally, local citations refer to the frequency with which a study is referenced by other researchers within the included articles. Global Citations, on the other hand, indicate the total number of times an article has been cited across all documents indexed in extensive bibliographic databases, such as Web of Science or Scopus. The data will be used to identify key publications and keywords in the research that require further investigation. Bibliometrix R package(https://github.com/massimoaria/bibliometrix) and VOSviewer were used to perform bibliometric analyses and visualization.

## Results

**Fig. 1:**
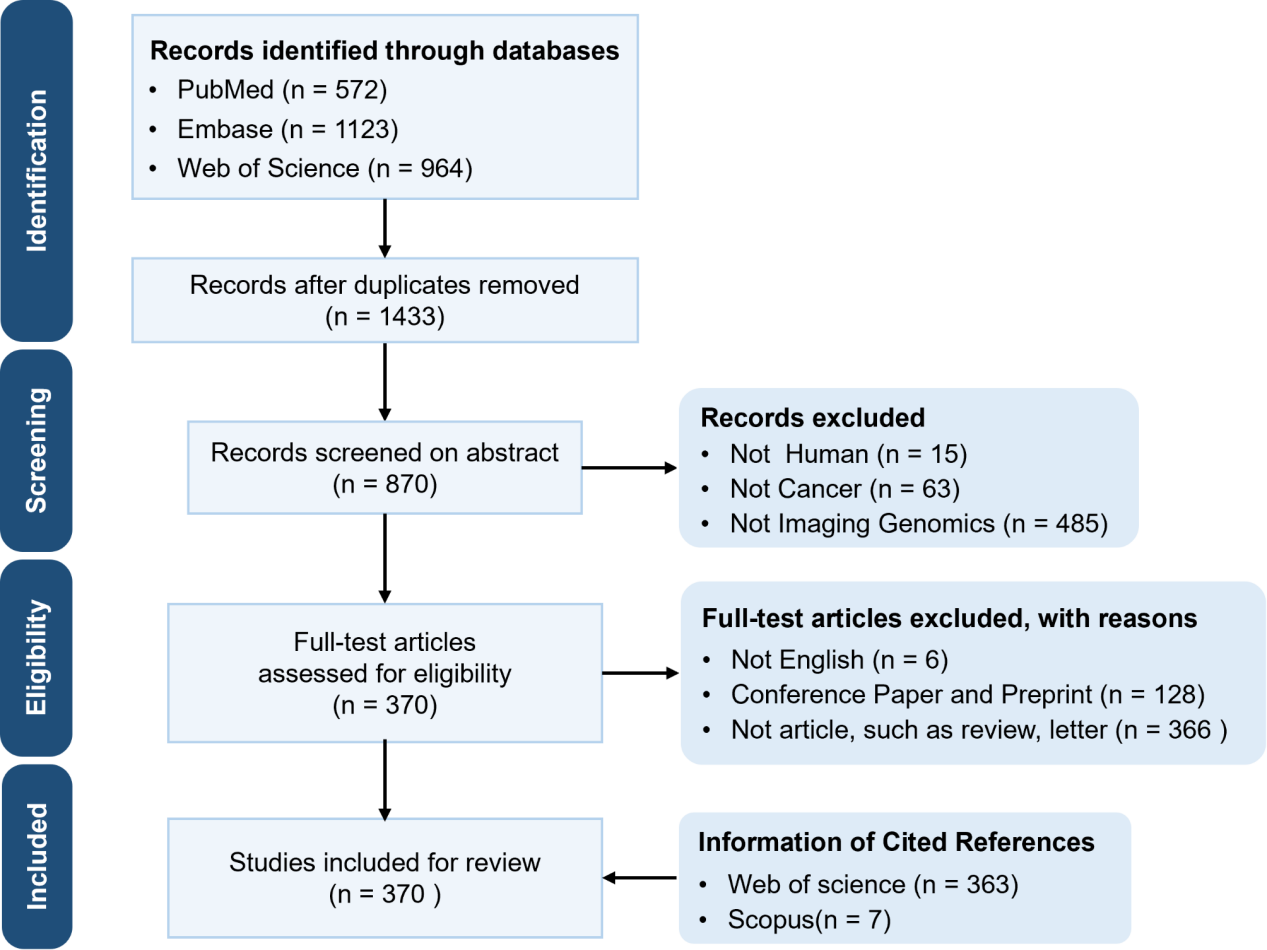
Schematic workflow of this review

Published literature was retrieved from PubMed (*n* = 572), Embase (*n* = 1123) and Web of Science (*n* = 964). Based on the inclusion and exclusion criteria, a total of 370 articles were selected for this review (Fig. 1).

### Annual publication output

From 2008 to June 2024, a total of 370 articles were published from 151 journals on the topic of cancer and imaging genomics. The data reveals a significant compound annual growth rate of 24.88%, indicating a rapidly increasing interest and research activity in this field. The internal co-authorship rate was 26.49%, with an average of 10.2 co-authors per document. A total of 10,528 references were cited across these publications.

### Affiliation and country

**Fig. 2:**
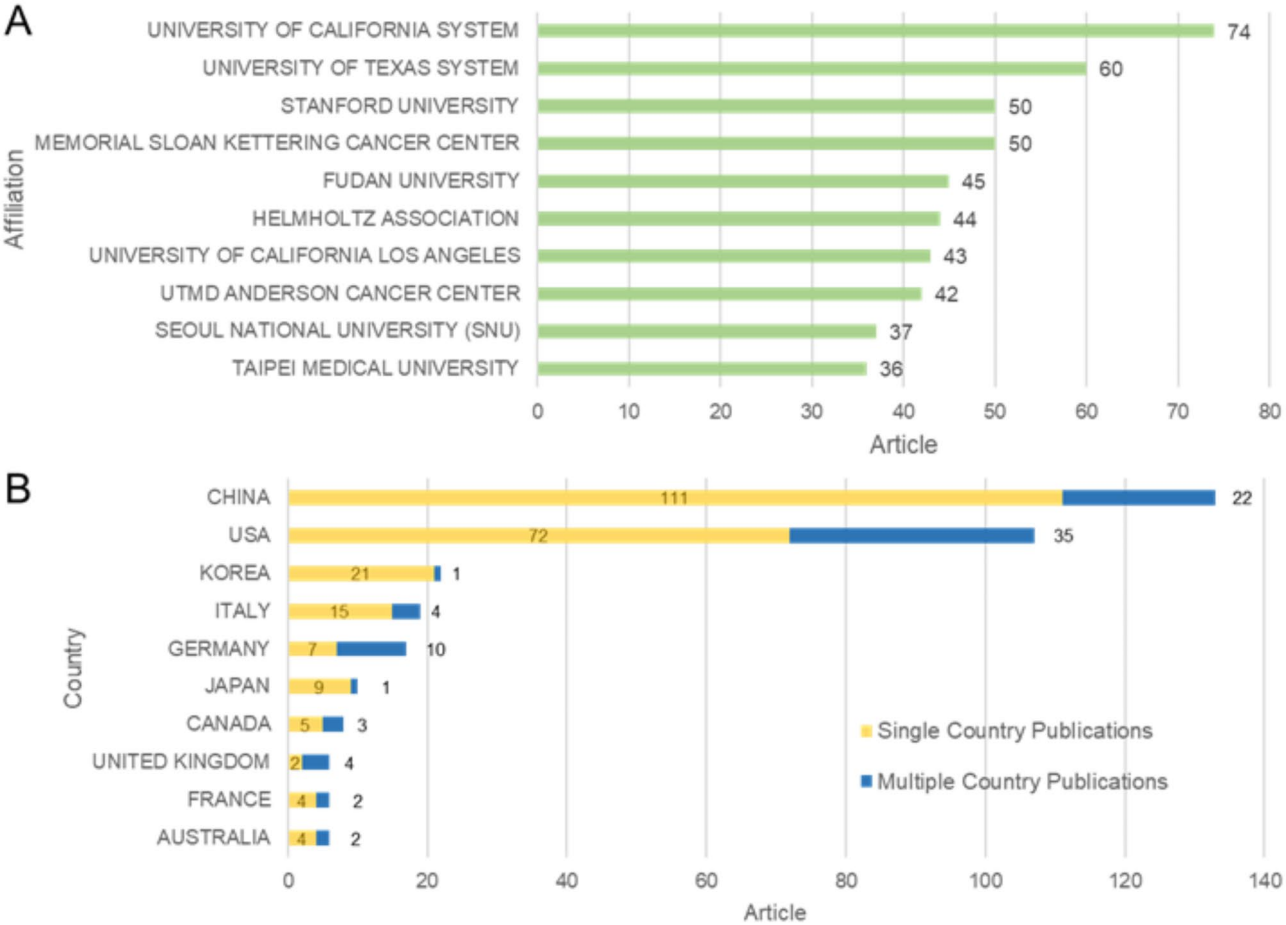
**A**, Bar chart showing the number of articles published by the top 10 Affiliations; **B**, Bar chart displaying the number of articles published by the top 10 Countries, with SCP representing Single Country Publications and MCP representing Multiple Country Publications

As shown in Fig. 2A, the most prolific source of published articles is the University of California, with a total of 74 publications. In second place, the University of Texas boasts 60 articles. Sharing the third position are the Memorial Sloan Kettering Cancer Center and Stanford University, both contributing 50 articles each. Fudan University secures the fifth spot with 45 articles, closely followed by the Helmholtz Association with 44 articles. From the data presented, it is clear that the University of California leads in research output. This likely reflects the institution’s strong research capabilities and extensive academic resources in the relevant fields.

After an analysis of corresponding author’s countries, it was exhibited that a total of 27 countries/regions in included articles. As shown in Fig. 2B, the country that contributed the largest volume of publications (133) was the China, which accounted for 35.9% of the total. USA was ranked the second (107, 28.9%), Korea third (22, 5.9%), and followed by Italy(19, 5.1%) and Germany (17, 4.6%). Multiple country publications (MCP) revealed the publication number of co-authors from different countries/regions. Although the USA had the highest MCP (35), its MCP ratio (= MCP/articles) was 32.7%. The top 3 cited countries were USA(5194), Netherlands(3336) and China(2339). Countries with close cooperation include USA and CHINA (22), USA and Germany (10), USA and United Kingdom (9). This indicates that the United States plays a significant role in international scientific research cooperation and maintains particularly frequent collaborative relationships with China. The collaboration between the two countries likely facilitates the exchange of research resources and technology, thereby enhancing their respective research capabilities.

### Source journals

In the bibliometric analysis of primary source journals, “Cancers” has emerged as the most prolific journal, with a total of 28 articles, underscoring its pivotal role in oncology research publication. It is closely followed by “Scientific Reports”, which has contributed a notable 20 articles, reflecting its multidisciplinary research focus. Ranking third is “European Radiology”, which has contributed 19 articles, highlighting its substantial impact in the field of medical imaging. “Frontiers in Oncology” and “Radiology” are tied for the fourth position, each journal contributing 16 articles, respectively.

In terms of the most locally cited sources, “Radiology” leads with an impressive 885 citations. “European Radiology” and “Neuro-Oncology” are the second most cited with 330 citations, followed by “Clinical Cancer Research” with 329 citations, and “Scientific Reports” with 314 citations. The most globally cited document is a paper authored by Hugo J. W. L. Aerts and colleagues, which was published in the journal *Nature Communications* in June 2014 and has garnered a significant global citation count of 3,281(Table 1) [30]. This research represents an early application of imaging genomics, examining CT scans from 1019 patients with lung and head and neck cancers across seven distinct cohorts [31]. The study aims to identify radiological characteristics that reflect intra-tumor heterogeneity and investigate the potential gene expression patterns associated with these features. As for the most locally cited document, it is a publication by GEVAERT O in the journal *Radiology* from 2012, with 33 local citations and a total of 310 global citations [32].

**Table 1:** The top 10 most globally cited documents in the imaging genomics

Paper	DOI	Total Citations	Total Citations per Year
AERTS H, 2014, NAT COMMUN	10.1038/ncomms5006	3281	273.42
WANG S, 2019, EUR RESPIR J	10.1183/13993003.00986-2018	274	39.14
GEVAERT O, 2012, RADIOLOGY	10.1148/radiol.12111607	310	22.14
KICKINGEREDER P, 2015, SCI REP-UK	10.1038/srep16238	231	21.00
KICKINGEREDER P, 2016, RADIOLOGY	10.1148/radiol.2016161382	208	20.80
DIEHN M, 2008, P NATL ACAD SCI USA	10.1073/pnas.0801279105	352	19.56
BUDA M, 2019, COMPUT BIOL MED	10.1016/j.compbiomed.2019.05.002	136	19.43
GEVAERT O, 2014, RADIOLOGY	10.1148/radiol.14131731	230	19.17
KARLO C, 2014, RADIOLOGY	10.1148/radiol.13130663	213	17.75
LIU X, 2018, NEUROIMAGE-CLIN	10.1016/j.nicl.2018.10.014	142	17.75

### Keyword occurrence analysis

**Fig. 3:**
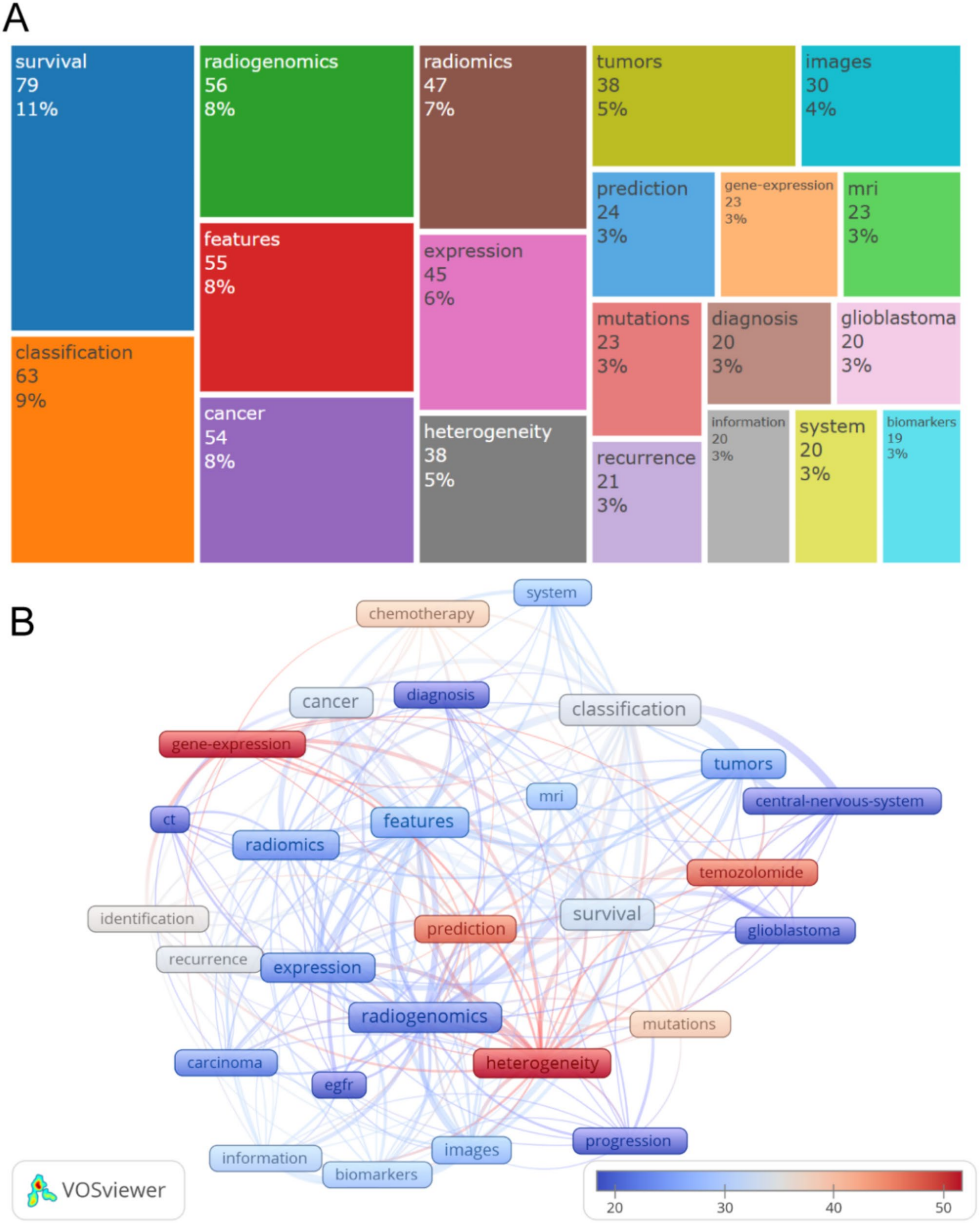
**A**, Treemap of top 20 Keywords plus; **B**, the average citation network map of the 28 keywords plus (node size represents the frequency of keyword occurrence in the included literature, and color indicates the average number of citations of documents associated with keywords)

A comprehensive collection of 919 unique keywords plus was assembled. Figure 3A illustrates the distribution of these keywords plus using a treemap visualization. The keyword plus “Survival” predominated with the highest frequency, appearing 79 times, indicating its significant role in the research field. It was closely trailed by “Classification” with 63 occurrences. These two high-frequency terms are interconnected and reflect the current research priorities.

Among the top 20 most frequently used keywords, ‘Glioblastoma’ was noted with 20 occurrences, standing out as the sole cancer type to make it onto the list, highlighting its particular relevance to the current body of research.

Moreover, Fig. 3B showed the citation network map of the top 20 keywords plus. The keywords plus of high-citation articles were as followed: “heterogeneity”, “gene-expression”, “temozolomide”, “prediction”, “mutations” and “chemotherapy”. These words indicate that current research primarily focuses on improving disease prediction accuracy, understanding tumor heterogeneity, exploring related gene expression patterns, and evaluating drug efficacy to optimize chemotherapy strategies. Among these, ‘temozolomide’ is a chemotherapy drug primarily used in the treatment of certain types of brain tumors. It achieves its therapeutic effect by methylating DNA, which inhibits the cells’ ability to replicate and grow.

**Fig. 4:**
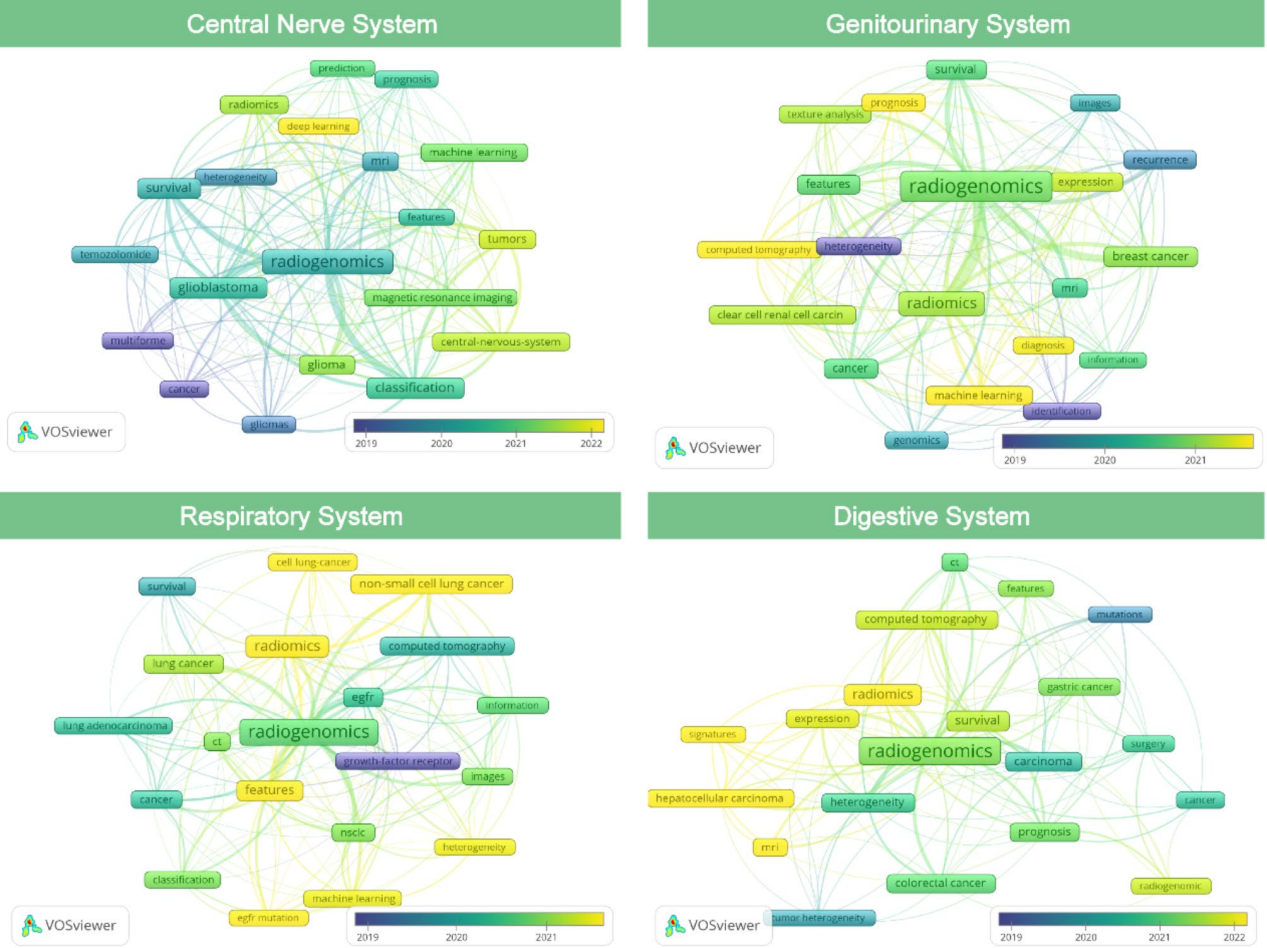
The network map of keywords in four main systems (node size represents the frequency of keyword occurrence in the included literature, and color indicates the publication years)

We conducted keywords and publication year analyses related to the central nervous system, genitourinary system, respiratory system, and digestive system (Fig. 4). In recent years, the main keyword was “deep learning” in the central nervous system, genitourinary system was “prognosis”, “diagnosis”, “machine learning”, “computed tomography”, respiratory system was “non-small cell lung cancer”, “cell lung cancer”, “features”, “egfr mutation”, “machine learning”, “heterogeneity”, and digestive system was “radiomics”, “hepatocellular carcinoma”, “MRI” and “signatures”. These keywords highlight the primary focuses within each system. “Deep learning” has become central in nervous system research, reflecting artificial intelligence’s rise in neuroimaging. Researchers use deep learning algorithms to enhance disease diagnosis, prognosis prediction, and treatment outcome evaluation. This technology improves accuracy and speeds up data extraction, offering tools for personalized medicine. In the genitourinary system, research aims to improve prognostic assessment and diagnostic precision. Combining “machine learning” and “computed tomography” enables more accurate lesion identification and personalized treatment plans. Predicting disease progression and treatment response remains a key focus. Respiratory system research emphasizes non-small cell lung cancer, particularly EGFR gene mutations. “Machine learning” technology helps researchers understand tumor heterogeneity, crucial for precise treatment strategies. In digestive system research, radiomics and hepatocellular carcinoma are prominent topics. Radiomics involves quantitative analysis of imaging data, revealing tumor structure and behavior. MRI supports precise diagnostics in the digestive system.

**Fig. 5:**
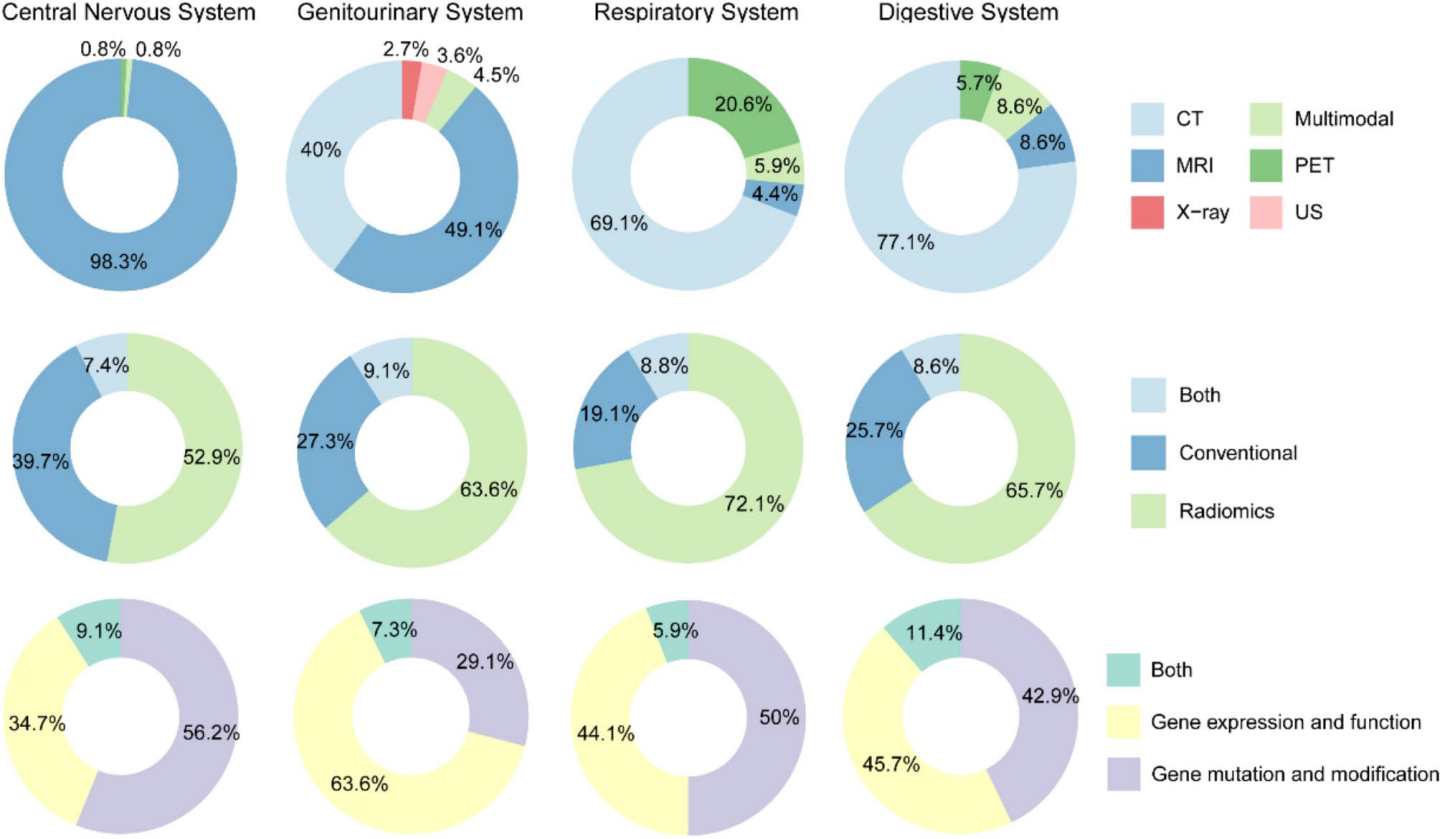
Pie charts illustrating the distribution of proportions for various imaging modalities, image features, and gene features across four major systems as reported in the included literature

The central Nervous System category included 121 articles, the genitourinary system had 110 (with breast cancer comprising 44 of these), the respiratory system accounted for 68 articles, the digestive system contributed 35, and the other systems category encompassed 36 articles (Fig. 5). The imaging modalities involved were as follows: 134 articles utilized CT, 193 employed MR, 28 used PET, 6 incorporated US, 3 applied mammography, and the remaining 18 articles featured multiple modalities. As depicted in Fig. 5, there is considerable variation in the imaging modalities used across different systems within imaging genomics. Notably, research pertaining to cancers of the central nervous system predominantly utilizes MRI (98.4%), whereas studies on cancers of the respiratory and digestive systems primarily employ CT scans. This disparity may be attributed to the development and application of imaging scanning technologies in different system.

Moreover, radiomics features have been the focus of 237 articles, taking the lead. Conventional imaging features are represented in 110 articles, and their combined application is discussed in 35 articles. Across various systems, articles utilizing radiomics features outnumber those employing conventional imaging features. In addition, studies examining gene expression and function were comparable in number to those investigating gene mutation and modification in both the respiratory and digestive systems.

## Discussion

### Current status of the research

The study analysis 370 articles about imaging genomics in cancer, which were from 2008 to 2024.

Imaging genomics, referred to radiogenomics, has become increasingly prevalent in cancer-related research, with a significant upward trend in publication volume over the year. On the one hand, public databases such as The Cancer Genome Atlas (TCGA) and The Cancer Imaging Archive (TCIA) provide crucial data support for the advancement of imaging genomics [33–35]. On the other hand, the advancement of artificial intelligence algorithms has enabled the extraction of high-throughput radiomics features and gene-related features [36–39].

This study meticulously analyzed and synthesized data from 370 included articles, which were from 2008 to 2024. The data revealed that the research has predominantly been concentrated in the United States and China, with close collaboration between the two nations, which is attributed to their innovative analytical techniques and extensive patient cohorts. Additionally, the research mainly focus on gliomas and breast cancer in this field [26, 33]. It may be because these cancer types have been extensively studied at the genomic level, and there is a growing body of evidence linking specific genomic alterations to imaging features. For gliomas, the emergence of novel advanced imaging techniques and the extensive work done by TCGA and the Ivy Glioblastoma Atlas Project on mapping genomic changes have led to the discovery of new correlations between genomic alterations and imaging features [40, 41]. Breast cancer, as the second common cancer type, has been found to be clinically and genomically heterogeneous, and incorporating genomic information into treatment decisions is a promising area of focus [1, 42, 43].

Bibliometric analysis reveals that “survival” and “classification” are the predominant keywords plus, underscoring the central themes in cancer imaging genomics research. “Survival” typically focuses on identifying features that may influence the prognosis of cancer patients, thereby further exploring related molecular mechanisms and predicting patient survival as well as recurrence and metastasis. A recent study analyzed multi-omics data from 1,754 glioma patients, identifying 35 genes and 82 radiomic features highly correlated [44]. This led to an effective predictive model for mortality risk and revealed associations with the tumor immune microenvironment. It indicates that genomics-imaging interaction can aid in accurately predicting glioma prognosis and immune responses. “Classification” centers on patient status, such as molecular subtyping and response to therapy. It involves investigating differences in imaging and/or gene features across different patient groups to explore the heterogeneity of cancers. It aids in establishing predictive and evaluative models, which can facilitate personalized medicine by tailoring treatments to individual patient characteristics. Furthermore, the primary objectives of current research were as follows:1) To investigate the relationship between imaging features and gene features; 2) To develop molecular typing and associated predictive models; 3) To establish models for predicting and assessing treatment outcomes; 4) To create risk stratification and prognostic prediction models.

### Basic workflow

**Fig. 6:**
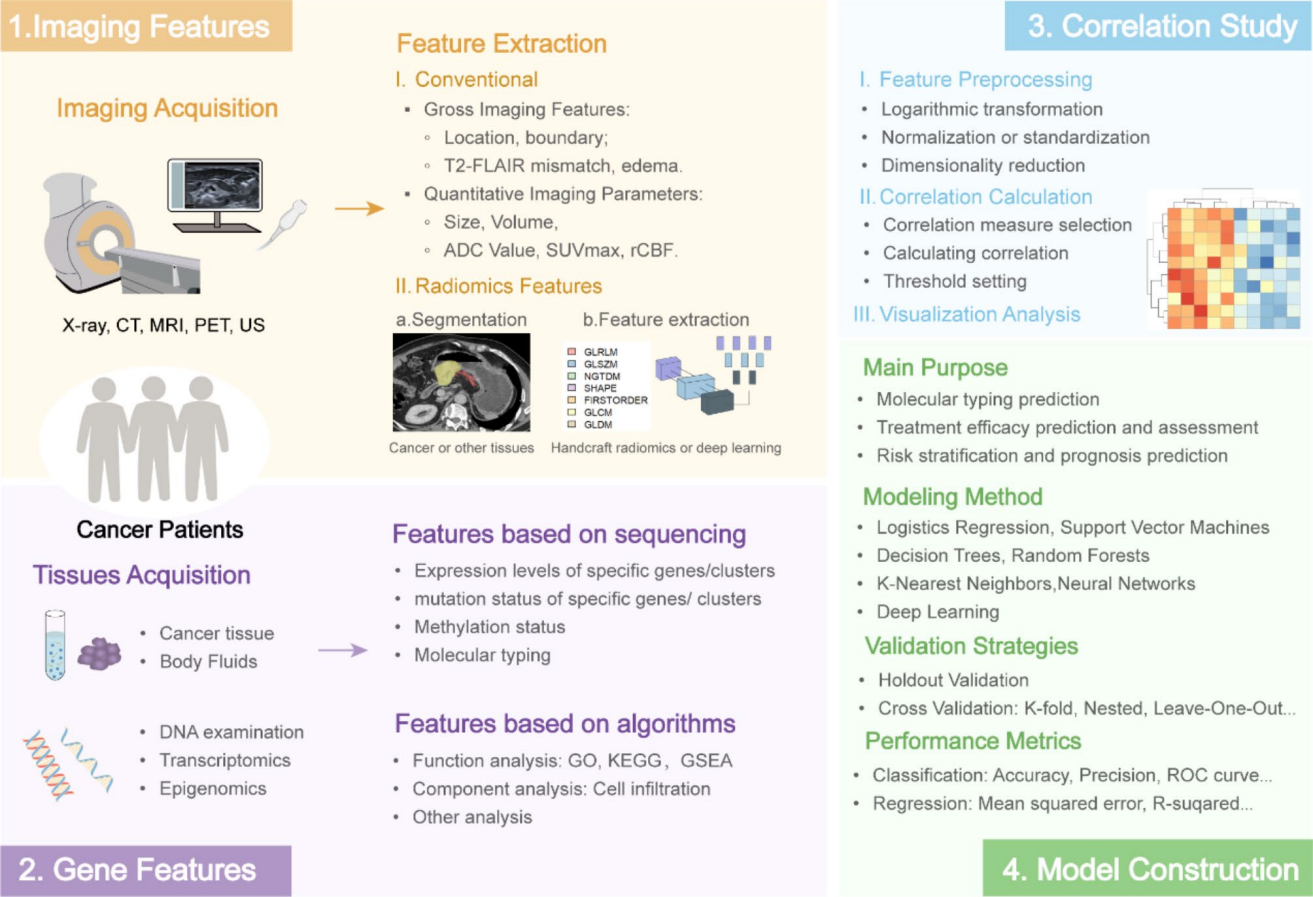
The basic workflow of Imaging genomics in cancer research

In cancer research, the fundamental workflow of imaging genomics can be segmented into four key components: data acquisition, imaging and gene features extraction, correlation analysis, and model construction (Fig. 6).

#### Imaging features

Imaging data can be provided by hospitals, institutions or public databases. The preeminent database for imaging genomics research is the Cancer Imaging Archive (TCIA), primarily because it merges TCGA to provide a comprehensive collection of clinical, genetic, and pathological information. Genomic data from TCGA is typically provided in several formats, including raw counts, TPMs (Transcripts Per Million), and FPKM (Fragments Per Kilobase of transcript per Million mapped reads). Moreover, the TCGA platform offers detailed information on mutation statuses and conducts foundational differential gene expression analysis. Common cancer imaging databases have be summarized by Piotr Woznicki et al. and Yusheng Guo et al. [35, 45].

Imaging features can be extracted after data preprocessing and segmentation. It can be categorized into two main types: conventional features(encompassing gross imaging features and quantitative imaging parameters) and radiomics features(which include both handcraft radiomics features and deep learning features). This study found that more than half of the included articles used radiomics features, which may be because radiomics features are large and relatively easy to obtain. Junior physicians can independently and relatively accurately outline lesions and tissues after a short training period. A large number of handcraft radiomics are available through Pyradiomics, HeterogeneityCAD or QIFE (Quantitative Imaging Feature Extraction) [46, 47]. They include first-order features(basic statistics of pixel intensity, second-order features(texture measures) and higher-order features. Moreover, select an appropriate deep learning framework to construct the deep learning model. Upon successful training of the model, one can proceed to extract deep learning features from different feature maps. These features are typically derived from intermediate layers within the model, especially from bottleneck layers, which are critical points where the network compresses and encodes essential information about the input data into a more abstract and condensed representation.

While some conventional features are obtained based on the rich clinical experience and summary validation of radiologists, the accurate assessment of some conventional features relies on the experience of radiologists, such as extramural vascular invasion score [48]. Because of the heterogeneity of cancer, prediction and assessment of molecular typing of cancer and its prognosis often requires more features. However, extracting a number of conventional features by senior radiologists is relatively time-consuming.

#### Gene features

Gene data can be obtained by whole genome sequencing, gene expression microarrays or quantitative PCR [49]. Currently, some gene features based on single-cell sequencing have also been applied to imaging genomics [45]. Common cancer-related gene databases include TCGA, Gene Expression Omnibus (GEO), and cBioPortal, which have DNA, transcriptomics, epigenomics or proteomics.

Imaging genomics research typically concentrates on analyzing gene expression, functionality, mutation, and modifications. The biological behavior of cancer is often too complex to be fully captured by the expression or mutation of a single gene [50]. Consequently, some imaging genomics research in cancer typically investigates alterations in multiple genes. In breast cancer, researchers frequently emphasize the OncotypeDX:21-gene Recurrence Score Assay, which can provide crucial insights into both the risk of recurrence and the potential efficacy of chemotherapy [51, 52]. Various analytical methods, including Gene Ontology, Kyoto Encyclopedia of Genes and Genomes, and Gene Set Enrichment Analysis, can be utilized to examine the functionality of genes or gene clusters. CIBERSORT is a computational tool designed for the analysis of immune cell composition in complex tissues using high-dimensional genomic data, particularly gene expression profiles from bulk tissue samples [53]. By estimating the abundance of immune and stromal cell populations in heterogeneous tissues, it can be used to estimate the relationship between cell subpopulations and treatment response and survival outcomes in cancer [54–56]. Furthermore, due to the transformative phase in genomics research, characterized by advanced bioinformatics technologies and AI integration, an increased amount of genetic and other biological information can now be extracted and analyzed with improved accuracy [45, 57].

#### Correlation study

In the field of imaging genomics research, the most prevalent correlation study involves identifying imaging features associated with genetic mutations, gene expression, or molecular tumor subtypes. Concurrently, certain studies investigate the molecular mechanisms underlying the appearance of imaging features. The research conducted by Yang Hao et al. examines the molecular mechanisms of CT-detected extramural venous invasion in gastric cancer, as well as its relationship with macrophages and cancer associated fibroblasts in the tumor microenvironment [58–60]. While the present study focuses on the utilization of radiomics features, it is conceivable that incorporating genomic analysis could enhance the understanding of the complex, high-dimensional radiomics features currently. Furthermore, some studies aim to identify image and genomic features associated with cancer patient prognosis or treatment efficacy [61–63]. These studies attempt to develop models using both image and genomic features. However, datasets containing paired image and gene information are typically limited in size.

#### Model construction

When assessing correlations and constructing models, it is paramount to tailor the analytical approach to the specific goals of the research and the distinctive attributes of the involved features. Deep learning models, such as Convolutional Neural Networks, demonstrate superior accuracy and performance in handling complex imaging tasks [64]. However, these models require large datasets to prevent overfitting and have high demands on computational resources, while their decision-making process is often opaque, which can be a drawback in clinical settings where transparency is crucial [43–45]. In contrast, simple models like logistic regression or decision trees perform well when data is scarce or computational resources are limited, and they offer greater interpretability.

However, they are less effective at capturing complex patterns in medical images. Therefore, model selection should account for task complexity, data volume, computational power, and interpretability.

### Challenges and future directions

Numerous studies have demonstrated the exceptional performances of these models. However, several challenges remain when considering the transition from theoretical models to practical clinical applications.

#### Ethical considerations of data

In the field of imaging genomics research, concerns about privacy and data protection are fundamental and highly significant [65, 66]. Organizations must navigate a complex landscape of regulations including General Data Protection Regulation and Health Insurance Portability and Accountability Act, which govern the use and sharing of genomic and imaging data. Adrien Oliva and colleagues have also outlined approaches and recommendations regarding the storage of genomic and health care data, privacy and security measures, and the management of informed consent [67].

Emerging technologies such as artificial intelligence (AI) raise additional ethical questions regarding bias in algorithms used for analysis or decision-making. This bias can stem from various sources, including data inequities and modeling choices. The development and validation processes of AI algorithms require transparency. Establishing clear definitions for fairness and accountability in AI systems is equally vital. As a result, relevant regulatory frameworks should adapt to the changing landscape and offer more comprehensive governance solutions.

#### Standardization in the research process

The challenge of feature standardization is well-documented, particularly within the realm of radiomics. Recently, guidelines such as the CheckList for Evaluation of Radiomics research (CLEAR) and the Radiomics Quality Score (RQS) approach have been published with the aim of improving the quality and rigor of radiomic research [8, 66, 68]. They covered study design, ethical considerations, data management, segmentation, preprocessing, feature extraction, and result dissemination. The variability in imaging equipment, including differences across manufacturers and scanning protocols, can influence imaging features [69]. As a result, data preprocessing is essential, and the selection of methods and parameter settings should be determined based on the specific condition. Certain researchers have compiled relevant guidelines [70, 71]. And the CLEAR checklist also recommends that studies related to radiomics should provide details on image preprocessing techniques [66]. In additions, to improve the consistency of conventional imaging feature extraction, the open-source ePAD platform (https://epad.stanford.edu) has been developed to facilitate quantitative imaging feature annotation and sharing [72]. For the consistency of radiomics feature extraction, medical imaging auto-segment is useful [73].

Similarly, gene features are susceptible to the impact of detection methodologies and bioinformatic analysis. The Association for Molecular Pathology, American Society of Clinical Oncology, and College of American Pathologists have jointly published the “Standards and Guidelines for the Interpretation and Reporting of Sequence Variants in Cancer,” which provides a consensus recommendation for the analysis and communication of genetic variations found in cancer contexts [74]. Moreover, Christina A. Austin-Tse and her colleagues contribute to the development of recommended protocols for clinical whole genome sequencing [75]. Additionally, certain analysis platforms offer standardized workflows. One such example is RaNA-Seq (https://ranaseq.eu/home), an accessible bioinformatics tool designed for swift RNA-Seq data analysis. This platform processes FASTQ files, generates quality control metrics, performs differential expression analyses, and aids in functional interpretation.

#### Model performance improvement

The model bias is mainly related to the characteristics and number of datasets used to train the model. Therefore, for Imaging genomics research, it is essential to have detailed patient inclusion and exclusion criteria and patient clinical characteristics. A large sample size is key to improving the model effectiveness and generalization ability. A large sample size required a large amount of manual segmentation work at the beginning. Existing automatic segmentation tools, such as MedSAM, MedSeg or MONAI, can be considered [76, 77]. However, these tools primarily focus on tissue or organ segmentation. Segmenting a part of the sample manually and training the automatic segmentation model to complete the subsequent segmentation can also be considered. To ensure accuracy, Dice scores need to be used to compare with the function obtained after manual segmentation, and radiologists also still need to check segmentation on a case-by-case basis [70]. And the establishment of databases containing medical segmentation, predictive modeling, etc. is important for the clinical translation of research results.

In addition, for most cases where sufficient data is not available, it is crucial to explore several strategies to enhance data availability and improve model performance. This includes fostering multi-center cooperation, which allows different institutions to collaborate and share their datasets. Furthermore, making full use of public databases can provide valuable supplementary datasets that may help fill gaps in local data availability. Employing sensible data augmentation techniques can also significantly enhance the diversity of the training dataset by artificially expanding it with variations of existing data. This approach helps models generalize better by exposing them to a wider range of scenarios. Researchers can also enhance deep learning model performance on limited datasets by using transfer learning and regularization methods [78–80]. Additionally, leveraging multimodal data—such as combining different imaging modality, gene-relevant information, proteomics and metabolomics—can provide a more comprehensive understanding. Similarly, incorporating time-series data can also capture dynamic changes in patient conditions over time, further enriching the dataset. Memorial Sloan Kettering Cancer Center has developed MSK-CHORD, a comprehensive dataset integrating natural language processing annotations with structured data on medications, demographics, and genomics from over 24,950 patients across various cancer types [81]. Machine learning models trained on this dataset have shown superior performance in predicting survival, and analysis of radiology reports within MSK-CHORD has revealed predictors of metastasis to specific organ. It demonstrated the significance of automated annotation of clinical notes and data integration to enhance predictions of patient outcomes.

#### AI integration for clinical translation

AI drives the advancement of imaging genomics in four key areas: automating the extraction of high-dimensional imaging and genomic features, facilitating the integration of multi-omics data, offering advanced model architectures, and enhancing the visualization of high-dimensional data to improve model interpretability.

As the results of this study show, the imaging features of existing studies are mainly radiomics features. Reducing time-consuming repetitive work through automated image preprocessing and automatic segmentation based on AI, completing radiomics feature extraction, and evaluating and predicting based on the constructed model will help accelerate its clinical application. Meanwhile, automated feature extraction also helps to reduce the subjective bias of manual outlining. And the enhanced interpretability of the model through gene-based validation and AI visualization algorithms (such as SHapley Additive exPlanations and Gradient-weighted Class Activation Mapping) will also help physicians understand the decision path of the model and enhance the acceptance of its application [82–84].

## Conclusions

Imaging genomics has made significant strides in cancer research, with a notable increase in publications and a focus on diseases like gliomas and breast cancer. Despite advancements, challenges in feature standardization and data variability persist, necessitating solutions like the CLEAR checklist and RQS for quality assurance. Moving forward, the field is set to benefit from enhanced collaboration, larger datasets, and the continued evolution of artificial intelligence in feature extraction.

## Data Availability

No datasets were generated or analysed during the current study.

## Abbreviations

AI: Artificial intelligence
CT: Computed Tomography
DWI: Diffusion Weighted Imaging
GEO: Gene Expression Omnibus
IHC: Immunohistochemistry
MCP: Multiple country publications
MRI: Magnetic Resonance Imaging
PET: Positron Emission Tomography
RQS: Radiomics Quality Score
SUVmax: Standardized Uptake Value max
TCGA: The Cancer Genome Atlas
TCIA: The Cancer Imaging Archive
TPM: Transcripts Per Million
US: Ultrasound
